# Assessment of Pharmacist’s Knowledge and Perception toward 3D Printing Technology as a Dispensing Method for Personalized Medicine and the Readiness for Implementation

**DOI:** 10.3390/pharmacy9010068

**Published:** 2021-03-23

**Authors:** Mohammed S. Algahtani

**Affiliations:** Department of Pharmaceutics, College of Pharmacy, Najran University, Najran 1988, Saudi Arabia; msalqahtane@nu.edu.sa; Tel.: +966-17-458-7200

**Keywords:** 3D printing, pharmacists, drug dispensing, knowledge, perception, implementation, personalized medicine

## Abstract

The main user of three dimensional (3D) printing for drug dispensing will be the hospital pharmacist. Yet despite the tremendous amount of research and industrial initiatives, there is no evaluation of the pharmacist’s knowledge and opinion of this technology. The present study aimed to assess knowledge and attitude among pharmacists about 3D printing technology as an innovative dispensing method for personalized medicine and the barriers to implementation in Saudi Arabia. We found that 53% of participants were aware of 3D printing technology in general, but only 14–16% of pharmacists were aware of the specific application of 3D printing in drug dispensing. Participants showed a positive perception regarding the concept of personalized medicine and that 3D printing could provide a promising solution to formulate and dispense personalized medicine in the pharmacy. It was also found that 67% of pharmacists were encouraged to adopt this new technology for drug dispensing, reflecting their willingness to learn new innovations. However, the technology cost, regulation, and the shortage of practicing pharmacists were also reported as the top barriers for implementation. Facilitating the implementation of this technology in the pharmacy practice will require a strategic plan in which pharmacists collaborate with regulatory bodies and 3D printing engineers to overcome challenges and barriers to implement such promising technology.

## 1. Introduction

Three-dimensional (3D) printing has paved a way forward through the technological paradigm shift by creating massive opportunities in diverse fields. The pharmaceutical field has faced slow innovation progress since the 1960s, and drug development procedures are time-consuming and require enormous resources [1]. Further, industrially produced drugs mostly fall under the principle of one-size-fits-all, where only a few dosage forms and strengths are produced, limiting the patient’s benefit and increasing associated side effects. Individual variation in drug response is due to the genetic and metabolic differences between patients [2]. This dilemma led to the emergence of the Precision Medicine Initiative in 2015 in the USA, which shifted the demand from mass production of medicine to personalized medicine. Personalized medicine tailors a drug dose for a patient based on their needs while increasing treatment efficacy and reducing side effects [3]. 

The recent innovation of 3D printing has presented immense prospects for revolutionizing the personalization of drug dispensing. The FDA approval of the levetiracetam (Spritam^®^) tablet in 2015 indirectly led to a tremendous increase in 3D printing research initiatives for the production of personalized medication and dose tailoring. Spritam^®^ is the first commercially available 3D printed drug; it is a highly porous tablet that disintegrates within seconds, and it is useful in epileptic seizures in adult and children [2].

Three-dimensional (3D) printing is the fabrication of a 3D structure into any shape and size by depositing materials layer-by-layer, and it is designed by computer-aided design (CAD) software. The current 3D printing technologies being investigated to fabricate medications vary from powder solidification (drop on solid, selective laser sintering), liquid solidification (stereolithography (SLA), microneedles, and drop on drop), and extrusion-based methods (fused deposition modeling (FDM) and extrusion at room temperature). The dominant techniques used are powder solidification and extrusion-based printing [2,4].

This technology can provide solutions to drug formulations that are not approachable by conventional manufacturing technologies. For example, the conventional fixed-strength production of drugs that have a narrow therapeutic index is commonly associated with fluctuating efficacy and toxicity. Three-dimensional (3D) printing can produce the exact dose needed by the patient, improving effectiveness and reducing toxicity [5]. Polypill is a pharmaceutical pill that contains a combination of drugs targeting a specific chronic disease such as hypertension and aims to reduce the number of tablets consumed by the patient to increase adherence to the therapeutic regimen. Shaban et al. successfully 3D printed a cardiac polypill consisting of five drugs, including an immediate-release compartment containing aspirin and hydrochlorothiazide, and a controlled release compartment containing atenolol, pravastatin, and ramipril. This ability to produce a complex system allows customization of the dose and release of each drug in the polypill system [6]. 

The Saudi Ministry of Health (MoH) established a new vision in 2012 aimed to improve the clinical pharmacy and pharmaceutical care services and reduce drug-related problems. This vision resulted in several initiatives that promote personalized medicine such as special pharmacy clinics that are run by specialized pharmacists. The anticoagulant clinic is an example of the special pharmacy where the pharmacists have the authority to adjust and change anticoagulant drugs when required. Other specialized pharmacy clinics include cardiology, solid organ transplant, pain, oncology, and infectious diseases [7]. The Saudi Food and Drug Authority (SFDA), which has the role to regulate food, drugs, and medical devices in Saudi Arabia, has issued general guidance for the use of 3D printing in the production of personalized medical devices. This guidance includes basic regulatory requirements needed to approve the use of 3D printing for the production of patient personalized medical devices [8]. We hope that the SFDA issue guidance and regulations specific for the use of 3D printing in the production of personalized medicine.

Even though the concept of 3D printing has been revolutionary, there are certain challenges for its implementation into the clinical pharmacy setting. The lack of knowledge and experience of 3D printing compared to the conventional manufacturing process poses several challenges, and many questions related to regulations, quality, and safety still remain. Implementing 3D printing in the pharmacy is complex and requires the development of effective strategies for effective outcomes. These implementation strategies require cooperation between practicing pharmacists, legislative bodies, and manufacturers.

The present study aimed to assess knowledge and attitude among pharmacists in Saudi Arabia about 3D printing technology as an innovative dispensing method for personalized medicine, and assess the readiness and possible barriers for implementation. 

## 2. Materials and Method

This observational cross-sectional designed survey was conducted to explore the knowledge and the attitude of the pharmacists working in Saudi hospitals toward the future use of 3D printing technique in the dispensing of personalized medicine. In addition, the study evaluates the readiness to implement such innovative technology in clinical practice. The questions were informed by reviewing the latest literature of 3D printing in the field of personalized medicine and previous surveys that studied the implementation of personalized medicine. The survey questionnaire was validated by a committee of three experts in the field.

### 2.1. Participants

The study sample consisted of 156 practicing pharmacists working in Saudi Arabia hospitals. Hospitals in Saudi Arabia are divided into three types: MoH hospitals, other governmental hospitals, and private hospitals. Participating in the survey was voluntary, and participants were free to leave at any point of the survey. The identity of the participants was kept anonymous with the full confidentiality of their responses. The agreement to participate in the survey was considered as consent. Approval of the study protocol was obtained from Najran University Research Ethical Committee on 20 January 2020 with Ref. No: 20-1-2-20 ET. 

### 2.2. Procedure

Google forms was used to construct and generate a hyperlink to the survey. Practicing pharmacists in Saudi hospitals were invited to participate in the study survey through the Twitter platform. This platform was used to invite the participants, as there was no accessible database to reach the pharmacists working in Saudi hospitals. Invitation messages were sent to users who identified themselves in their account bio as a hospital pharmacist working in a Saudi hospital. Then, the invited pharmacists were asked to distribute the survey among their colleagues in the hospital.

The invitation message consisted of the 3D printing definition, the study objective, survey hyperlink, and a video showing the process of fabrication of a dosage form using a 3D printer. The survey was open for participation between 10 May through 4 June 2020. 

### 2.3. Materials

The survey consisted of 32 questions divided into five sections as following:

First section: Socio-demographic profile. Pharmacists were asked about their educational and professional qualifications, the type of hospital they work in, and the number of years since completing their latest degree. 

Second section: Each pharmacist’s knowledge was evaluated regarding the 3D printing technology and their applications in the medical and the pharmaceutical field.

Third section: Participants were asked about their perception on the extent to which a patient’s genetic profile affects their response to drugs, the need for more personalization of medicine, and if the personalization of medicine will enhance patient adherence to the treatment. 

Section four: Pharmacists were asked about their perception regarding the future use of 3D printing as an innovative method of drug dispensing. This perception was evaluated in terms of the role of 3D printing in increasing the efficacy and safety of medication and the possibility of producing different dosages and dosage forms, and the effect of implementing 3D printing on pharmacy workflow.

Section five: Participants were asked about the availability of automated drug dispensing systems and the availability of 3D printing in their institution for any medical application and their support of implementing 3D printing in the pharmacy. At the end of the questionnaire, the participants were asked about the expected barriers that might limit the implementation of 3D printing.

### 2.4. Data Analysis and Statistics

Statistical Package for Social Sciences (SPSS) software (ver. 23; IBM SPSS Inc., Chicago, IL, USA) was used for the statistical analysis. The Chi-square test was used to assess associations between categorical variables. Statistical significance was set at *p* < 0.05. 

## 3. Results

### 3.1. Socio-Demographic Characteristics of Study Participants

Out of the approximately 1100 hospital pharmacists invited to participate in this survey, 156 (14%) pharmacists participated in the survey. Eighty pharmacists served in MoH hospitals and represent half of the participants in this study. Almost 40% of the participants work in other governmental hospitals, while less than 10% work in the private hospitals. The majority of the study participants (107, 69.5%) were designated as a Pharmacists, while 38 (24.3%) and 11 (7%) were designated as Senior Pharmacists and Consultant Pharmacists, respectively. More than one-third of the participants (56, 35.8%) had administrative tasks. These designations are given to the medical professionals by the SCHS based on the academic degree, experience, and other requirements based on the medical specialty. In general, medical professionals are prohibited from practice in health facilities unless registered and classified by SCHS.

The majority of the study participants held a bachelors’ degree; the minimum qualification to practice pharmacy (64, 41.6%), which was followed by 39 (25.3%) holding a masters’ degree. The remaining (36, 23.4%) held a Pharm D degree, and very few had a PhD degree (15, 9.7%). Thirty-six (23.4%) had recently (less than two years from the time of survey) completed their last educational degree, and 23 (14.9%) completed their educational degree 2 to 4 years before the survey. Thirty-nine (25.3%) study participants had completed their last educational degree in the last 5 to 7 years, and 31 (20.1%) had completed them in the last 8 to 10 years. In addition, twenty-five (16.2%) of the participants received their last educational degree over 10 years ago. The majority of the participants (93, 60.3%) were between 2 and 10 years since their last educational degree (Table 1).

### 3.2. Knowledge about 3D Printing Technology 

**Figure 1:**
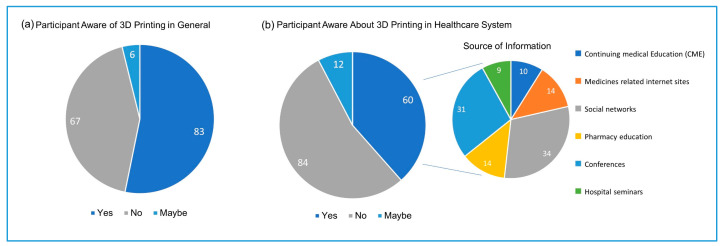
Participant’s awareness of three dimensional (3D) printing in general and in the healthcare system. Source of information (*n* = 60, multiple response by each participants).

While 83 (53.2%) study participants were aware of 3D printing technology in general (Figure 1a), only 60 (38.5%) knew of the existence of it in the healthcare system (Figure 1b). Based on the participants designation, the awareness was higher among Pharmacists, *n* = 38 (24.4%) followed by Senior Pharmacists, *n* = 16 (10.3%) and Consultant Pharmacists *n* = 6 (3.8%); however, this finding was statistically not significant (*p =* 0.842). The main source of awareness about 3D printing in the healthcare system is social networks and scientific conferences, followed by pharmacy education and medicine-related websites. Continuing medical education (CME) and hospital seminars were the least frequent source of information about 3D printing (Figure 1b).

Even a lesser proportion had heard about the future use of 3D printing technology in the pharmaceutical sector; less than one-quarter of the participants (35, 22.4%) knew that 3D printing technology can produce a dosage form with a precise dose, and only 27, 17.3% knew about the ability of the same technology to produce a dosage form with customized drug release. Based on the participants’ designation, the knowledge was higher among Pharmacists (22, 14.1%) followed by Senior Pharmacists (10, 6.4%) and Consultant Pharmacists (3, 1.9%). This finding was statistically significant (*p* = 0.690). Furthermore, based on the participants’ educational degree, pharmacists with an M.Sc. degree were having marginally higher knowledge (12, 7.7%) than B.Sc. (10, 6.4%) followed by Ph.D. (7, 4.5%). These trends were statistically significant (*p =* 0.045). Participants who have completed their last degree 5–7 years and 8–10 years ago were having higher knowledge than participants who has obtained their degree in last 4 years (*p =* 0.219), and this is statistically not significant. Only 22, 14% out of 156 participants were aware of the FDA-approved product (Spritam^®^) manufactured using 3D printing technology. 

More than 50% of participants were aware of 3D printing technology in general and in healthcare, but only 14–16% were aware about the specific application of 3D printing in drug dispensing.

### 3.3. Perception about Personalized Medicine

**Figure 2:**
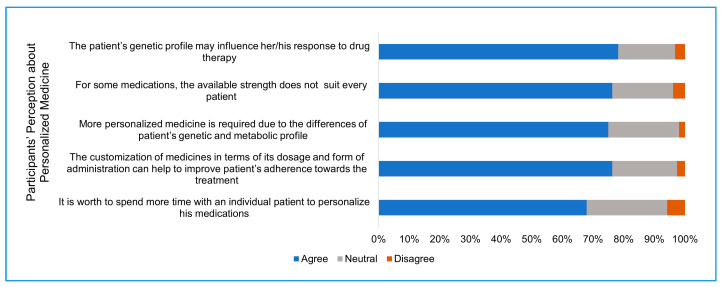
Participants’ perceptions about personalized medicine.

The participants’ perceptions regarding the importance of personalized medicine was evaluated. The ability of 3D printing to produce a precise dose and customized drug release in a small scale makes it a promising method to produce personalized treatment based on the individual medical profile. Positive perception was received from the participants’ responses about the importance of personalized medicine (Figure 2). The majority of the participants (122, 78%) agreed that the patient’s genetic profile may affect their response to a drug therapy, while 29 (18.5%) had a neutral response and 5 (3.2%) disagreed. Additionally, there was a similar response from participants to the statement that the available dose strength for some medications does not suit every patient. Three quarters of the participants (117, 75%) agreed that more personalized medicine is required to meet the differences in patient’s genetic and metabolic profiles, while nearly one-quarter (36, 23%) were neutral about it, and only 3 (2%) were against the need for more personalized medicine. In addition, more than three-quarters of the participants (119, 76%) agreed that the customization of the dosage form can help improve the patient compliance to the treatment, while one-fifth (33, 21%) had a neutral response and 4, 2.5% were against it. Sixty-seven percent of the participants (106 out of 156) agreed that it is worthwhile to spend more time with individual patients to personalize his medication, while around one-quarter (41, 26.2%) had a neutral response to this statement, and 9 (5.8%) were against it. 

### 3.4. Participants’ Expectations toward 3D Printing as a Method of Drug Dispensing

**Figure 3:**
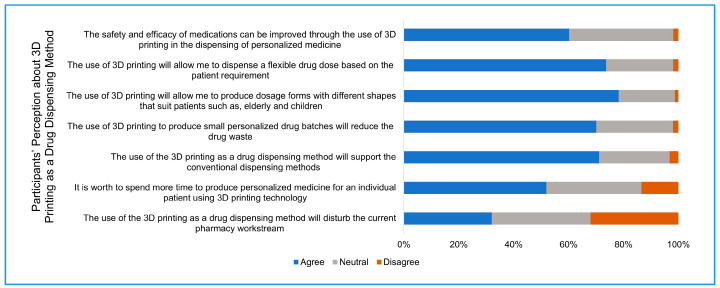
Participants’ perceptions of the application of 3D printing as a novel drug dispensing method for personalized medicine.

Based on participants’ knowledge, the survey introduction, and the presented video clip that explains the printing process of the dosage form, the participant’s expectations about the future application of 3D printing as a drug dispensing technology were recorded. Participants generally have a positive expectation about the promising advantages of 3D printing to produce personalized medicine (Figure 3). Around 60% of the participants (94 out of 156) agreed that the safety and efficacy of medications can be improved by dispensing personalized medicine using 3D printing, while 59, 37.8% had a neutral response to this claim, and less than 2% (3 out of 156) were against this claim. Around three-quarters of the participants (115, 73.7%) agreed that the use of 3D printing would allow dispensing flexible doses to suit the patient requirements, while around one-quarter (38, 24.3%) had a neutral response, and less than 2% (3 out of 156) disagreed with this statement. Furthermore, more than three-quarters of the participants (122, 78.2%) were in favor of 3D printing to provide the opportunity to produce different shapes of dosage forms to suit patients such as the elderly and children, while one-fifth of the participants (32, 20.5%) had a neutral response to this expectation, and only two participants only disagreed entirely. Almost 70% of the participants (108 out of 156) agreed that the ability of 3D printing to produce small drug batches will reduce drug waste, while 43 (27.5%) had a neutral response, and only three were against this statement. Nearly three-quarters of the participants (111, 71%) expected that the use of 3D printing as a drug dispensing method would support the conventional drug dispensing methods, while one-quarter of the participants do not expect that 3D printing will support the conventional dispensing method, and five participants disagreed. Almost one-third of the participants agreed (50 out of 156) expect that the use of 3D printing as a drug dispensing method will disturb the current pharmacy workstream. The same number of participants expect that the use of 3D printing will not disturb the workstream, and 56 (35.9%) had a neutral response to this expectation. Around 60% of participants (81 out of 156) supported spending more time to produce personalized medicine for individual patients using 3D printing, while 54 (34.6%) had a neutral response, and 21 (13.4%) disagreed with this statement (Figure 3).

### 3.5. Implementation of 3D Printing Technology in Healthcare Practice

In recent decades, various advanced technologies have been used for drug dispensing inside hospitals. These technologies have helped accelerate the process of drug dispensing and reduce associated human error, resulting in more accurate dispensing. The introduction of such technologies into the pharmacy practice reflect the willingness of the hospital and pharmacy administration to accommodate new technologies for the benefit of the patient. Here, we measure the distribution of automated drug dispensing technologies among the participants’ hospitals. The automated intravenous (I.V) compounding system is available at 48 (30.7%) of the participants’ hospitals, while 85 (54.4%) reported it as not available, and 23 (14.7%) were unsure. Furthermore, the automated unit dose system is available at 47 (30.1%) of the participants’ hospitals, while 94 (60.2%) reported it as not available at their hospitals, and 15 (9.6%) were unsure. The participants were asked if 3D printing technology is available for any clinical application at their hospitals. Only 8 (5%) of the participants confirmed the availability of such technology, while three-quarters (117, 75%) reported that it was unavailable, and 31 (19.8%) were unsure. Two participants had cited the utilization of 3D printing technology in the field of research, while one participant cited its practical application in preparing a mask for the patient who was to receive localized radiation as treatment for melanoma. 

**Figure 4:**
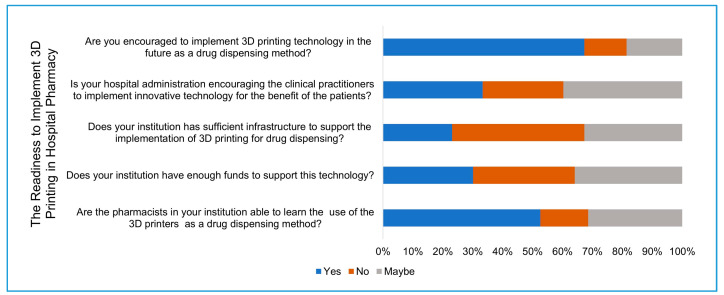
The readiness of hospital pharmacies to accommodate 3D printing technology for drug dispensing.

The readiness of the participants as pharmacists and their institutions to implement 3D printing as a part of the pharmacy practice were evaluated (Figure 4). Participants had encouraging attitudes for implementing such technology for drug dispensing (105, 67.3%), while only 22 (14.1%) were not encouraged, and the rest were unsure. One-third of the participants confirmed that their hospital administration encourages the clinical practitioner to implement innovative technologies for the benefit of the patients (52, 33.3%), while around one-quarter of the participants (42, 26.9%) felt that their hospital did not encourage the implementation of innovative technologies for the benefit of the patients, and 62 (39.7%) were unsure. Less than one-quarter of participants believed that their institution had sufficient infrastructure to implement 3D printing in the pharmacy practice (36, 23%), while 69 (44.2%) did not think that and 51 (32.6%) were unsure. In addition, 47 (30.1%) of the participants believe that their institution has enough funds to implement such technology in the pharmacy practice, while 53 (34%) did not believe that, and 56 (35.9%) were unsure. More than half of the participants (82, 52.6%) confirm that their colleagues in the pharmacy are able to learn the use of the 3D printing technology for drug dispensing, while only 25 (16%) of the participants did not confirm that, and 49 (31.4%) were unsure (Figure 4). 

### 3.6. Possible Barriers to Implement 3D Printing in the Pharmacy Practice

**Figure 5:**
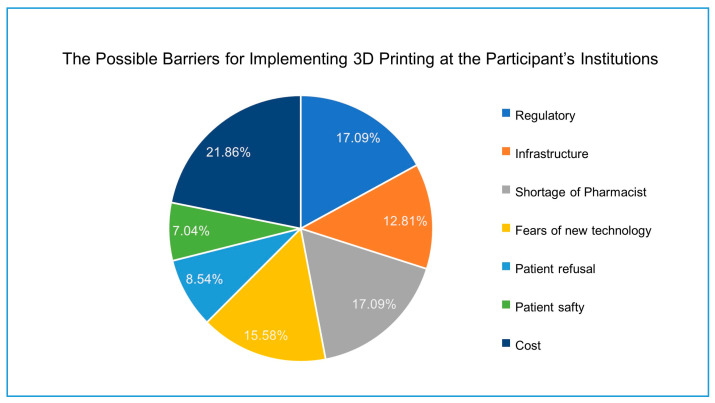
The main possible barriers that can affect the implementation of 3D printing in the participants’ institutions. (*n* = 156; multiple responses by each participant).

Participants were asked for possible barriers affecting the implementation of 3D printing at their institutions (Figure 5). Cost represented the major barrier (21.8%), followed by regulatory (17.1%), shortage of pharmacists (17.1%), fears of new technology (15.58%), and infrastructure (12.81%). The least barriers selected were patient refusal (8.54%) and patient safety (7.04%).

## 4. Discussion

This study was designed to evaluate the hospital pharmacist’s knowledge about 3D printing technology and their expectations from this technology as a new method of drug dispensing in pharmacy practice. Three-dimensional (3D) printing has made a big impact in many areas of life, including engineering, educational, and clinical applications [9]. Pharmaceutical research has been predicted to significantly benefit from 3D printing in the field of drug production. This research has proven that this technology can produce a drug in personalized doses and customize drug releases. Three-dimensional (3D) printing is a promising solution to personalized medication to provide better therapeutic efficacy and less adverse effects [4]. Despite the tremendous number of studies testing 3D printing technology for the future use in drug dispensing, the pharmacist is still disconnected from this progress; hence, this study was conducted. 

The common trend globally is the transformation from general pharmaceutical services to more customized clinical and specialized pharmaceutical care. Pharmacy practice in Saudi is no exception, as it is well-established and has dramatically improved in recent years with accreditation from the Council for Pharmacy Education and the American Society of Health-System Pharmacists [10]. Several initiatives and practices have been introduced into pharmaceutical services to promote medication therapy management, such as electronic prescriptions, automated dispensing systems, and automated I.V. compounding systems. In addition, there has been an initiation of special pharmacy clinics managed by specialized pharmacists to enhance the treatment efficiency and reduce associated medication errors through a cost-effective approach. Anticoagulant clinics are an example of specialized pharmacies managed by pharmacists with the authority to adjust doses, change medications, and add other anticoagulants when needed. In addition, other specialized pharmacies such as cardiology, solid organ transplant, pain, and oncology have been implemented in several Saudi hospitals [7]. All of these advanced practices reflect the government’s keenness to improve pharmaceutical services, which suggests that innovative methods such as drug dispensing using 3D printing technology may be one of the future practices in this sector. 

The FDA approval of the first drug (Spritam^®^) produced by 3D printing in 2015 stimulated the enthusiasm to explore the possibility of using this technology in drug dispensing. Despite the high trend of 3D printing in recent years, only 38% of the study participants were aware of the using of this technology in healthcare, and less (22%) were aware that this technology can produce drugs in precise doses. Only 22 out of 156 participants were aware of Spritam^®^. Pharmacy education was the source of this awareness of 3D printing use in the healthcare system to only 14 participants, indicating that the traditional pharmacy education in Saudi is lagging behind the trends in the pharmaceutical research. Participants indicated that the main source of awareness about the 3D printing applications in the healthcare systems is the social networks, followed by scientific conferences. Social media has provided a unique opportunity to share new research and clinical guidelines that were once only available through scientific conferences and journals or professional organizations. A systemic review conducted by Benetoli et al. found that the social networks have facilitated professional communications and interaction between the healthcare providers [11]. Therefore, pharmaceutical researchers and pharmacists are encouraged to share knowledge and communicate recent advances in research and practices through social media guided by professionalism and ethics. Conferences were selected by the participants as the second source of awareness about the use of 3D printing in the healthcare system. 

The common practice in pharmaceutical industries is to produce a few discrete drug strengths and forms for all consumers. However, individual variability related to genetic, ethnic, gender, age, and weight makes the concept of one-size-fits-all challenging to achieve in real practice, and some consumers will be exposed to high doses and more side effects and the others exposed to under-therapeutic doses. The idea of personalized medicine has grown dramatically in recent years where dose adjustments are made according to pharmacokinetic and pharmacogenetic profiles of the patient at the point of care in order to improve the efficacy and to reduce the toxicity of the drug. The flexibility of 3D printing to produce a small scale of customized doses and release profiles provides a promising solution to implement personalized medicine practice in the healthcare sector [12]. The personalization of medicine has been implemented to some extent by pharmaceutical services in Saudi hospitals, such as in the anticoagulant clinic where the doses or drugs are adjusted frequently based on the patient blood profile [7]. Participants of this study had a positive perception about the concept of personalized medicine. A similar percentage of respondents believe in the need for more drug personalization and believe that it will increase the patient’s commitment to his treatment plan. Interestingly, a high percentage of participants (76%) are willing to spend more time to personalize treatment for individual patients. This additional time could be used to prepare and set up the 3D printer to fabricate the individual patient drug batch. The high perception presented by pharmacists working in Saudi hospitals about the importance of pharmacogenomics [13], in addition to the high perception given at this study about the importance of personalized medicine, increases the future role of 3D printing in the production of effective and safe medicine.

Three-dimensional (3D) printing presents a promising solution to tailor dosage forms for specific doses and release profiles. Various startup and pharmaceutical companies have started prototyping 3D printers and automated systems specific for the production of pharmaceutical dosage forms. For example, FabRx is a startup biotech company specializing in 3D printing of medicine that managed to bring the first pharmaceutical 3D printer named M3DIMAKER™. This printer is specialized with a hardware that can use different printing nozzles and software to allow the selection of the necessary dose by the pharmacist based on the given prescription [14]. In this study, participants had high positive expectations about the future application of 3D printing in the pharmacy practice. More than 60% of the participants expected that the use of 3D printing will improve the efficacy and safety of medications, and around three-quarters of participants expected that this technology will give the opportunity to produce flexible dosage forms. 

Drug waste has a great negative impact on the economy and the environment. The World Health Organization (WHO) has reported that half of the medications dispensed are sold improperly, and half of the patients do not adhere to their treatment plan [15]. Faten Alhomoud conducted a survey to study the waste-reducing activities among the practicing pharmacists in the Gulf region countries. Pharmacists participating in this study believe in the importance of reducing drug-waste; however, waste-reducing activities were not practiced continuously [16]. A study found that on average, 2–3 drugs are expired per household in Saudi [17]. The ability of 3D printing to produce personalized medications on a small scale, and the concept of a polypill provide a great solution to reduce the drug waste [12]. 

The future of 3D printing in pharmacy practice focuses on dispensing the personalized medicine where specific doses, drug release, or dosage form shape is required. Therefore, this technology is designed to support and not to replace the current practice of dispensing the mass produced medicines by the pharmaceutical industries [2]. Around 70% of the participants agreed that the use of 3D printing as a drug dispensing method will support the current conventional method of drug dispensing. On the other hand, more than 30% of the participants believed that the introduction of this technology will disturb the current workstream in the pharmacy. Therefore, careful planning is needed to implement this technology into the pharmacy practice with minimal disruption to the current workflow, and it will include technical and legislative considerations, infrastructure changes, and training practicing pharmacists for 3D printing [18]. Automation in drug dispensing has increased globally to improve dispensing accuracy and to reduce human error, as well as to curb the effect of labor cost [19]. Additionally, automation has allowed the pharmacist to perform more valued patient care practices such as patient counseling and drug monitoring [20]. 

The automated unit dose systems and IV compounding system are examples of automation in pharmacy practice. Thirty percent of the participants stated that their hospitals have automatic unit dose systems, and around the same percentage stated that their hospitals have the I.V. compounding system. The implementation of such technologies into the pharmacy practice reflect the willingness of the hospital and pharmacy administration to accommodate new technologies for the benefit of the patient. Since the early 2000s, 3D printing technology has been applied in different medical fields such as dental implants and prosthetics. The applications have expanded considerably to cover several specialties including tissue and organ bioprinting, anatomical models for complex surgery interventions, drug delivery, and personalized implants [21]. At this study, only eight participants reported the use of 3D printing at their hospitals. 

The role of hospital administration has also expanded widely as healthcare systems continuously innovate and develop to respond to the changes in diseases, the impact of aging populations, and the advances in diagnosis and treatments [22]. Lack of support from healthcare leaders has been identified as one of the major barriers of implementing new technologies [23]. Despite the large percentage (67%) of participants who support the implementation of 3D printing, only 33.3% of them believe that their hospital administration is supportive of innovative technologies and encourages clinical practitioners to implement them. Furthermore, more than half of the study participants believe that their colleagues are able to learn the process of 3D printing for drug dispensing. Even so, only 30% of the participants believe that their hospitals have sufficient infrastructure to implement 3D printing, and less than 30% of the participants believe that their hospitals have the sufficient financial support to implement this technology.

The Expert Recommendations for Implementing Change (ERIC) project has provided a consensus of 73 discrete implementation strategies for innovative technologies. Those strategies are used to enhance the adoption, the sustainability, and the spread of an innovation. Assessing the readiness and identifying possible barriers is an implementation strategy and is particularly useful before the formal implementation [24]. The survey participants assessed the possible barriers that might affect the implementation of 3D printing for drug dispensing. The cost of the technology implementation was selected as the greatest barrier to overcome. Three-dimensional (3D) printing has proven cost-effective, especially for small-scale production. Therefore, 3D printing has a great potential to reduce medical cost. In surgery practice, 3D printing of anatomical models helps the surgeons simulate the surgery to reduce the surgery time, complications, and ultimately reduce the surgery overall cost. The opportunity of producing personalized medicine and polypill formulations will improve the patient compliance and reduce the material costs [25]. The collaborative effort between the healthcare providers and 3D printer manufacturers, the availability of the printing materials, and the expansion of manufacturing of 3D printing systems designed for medical applications are factors that will reduce the cost of implementation [26]. 

Staff shortage was selected by the participants as a barrier for the future implementation of the 3D printing in practice after the cost. The transformation of the general pharmacy practice to a specialized pharmacy practice with more determination on patient counselling and drug monitoring increases the demand for more qualified pharmacists. Albekairy et al. evaluated the sufficiency of the clinical pharmacists in the National Guard Affairs central region hospital, Saudi Arabia. The study indicated that the number of clinical pharmacists is not enough to perform the clinical pharmacy services; the total number of practicing clinical pharmacist was 24 at the time of the study, while the study suggests adding 60 to 65 positions to adequately staff a clinical pharmacy [27]. The shortage of trained pharmacists is a major reason hindering most pharmacy initiatives or services. 

Regulations of pharmacy practice is another possible barrier for the future implementation of 3D printing in pharmacy. The general administration of pharmaceutical care in the MoH and the SFDA are the regulatory bodies for the pharmaceutical services, including clinical pharmacy practice and clinical pharmacy programs. Since 2013, the general administration of pharmaceutical care has implemented a national plan with new set of standards and regulations to meet all the American Society of Health-System Pharmacists (ASHP) standards and regulations [28]. The application of 3D printing in the medical field is still in its infancy; however, the FDA has been the forerunner on implementation and has issued technical guidance for the additive manufacturing of medical devices on December 2017. The guidance is consists of two sections: Design and manufacturing considerations, and printed device testing considerations [29]. The FDA Drug Evaluation and Research approved Spritam^®^ as the first 3D-printed drug in 2015. This medicine complies with the existing chemistry, manufacturing, and control standards as any other solid dosage form [30]. The uniqueness of the 3D printing process compared to the conventional drug production and the lack of previous clinical experience raises challenges to regulate such practice. One of the challenges is the quality assurance of 3D-printed dosage forms. The current traditional methods used to test the quality of a conventional tablet including the content uniformity, disintegration, hardness, and dissolution test will vary when applied to the 3D printed formulations. In addition, such tests are destructive and impractical in the clinical sitting. Therefore, revised quality tests need to be issued by the FDA and pharmacopoeias to be more applicable for the 3D printed formulations [12].

Fear of new technology is the next barrier for the future implementation of 3D printing for drug dispensing. Understandably, most of the practicing pharmacists have reservations with any change that can affect the pharmaceutical services workflow. Fear of implementing new technologies such as 3D printing may restrict or slow the development of pharmacy services [31].

Patient safety and patient refusal were the lowest barriers for the implementation of 3D printing. However, a study by Goyanes et al. conducted a study in 2017 among pediatric patients to evaluate isoleucine blood levels after six months of treatment with two types of formulations: conventional capsules prepared by manual compounding, and personalized chewable formulations prepared by automated 3D printing. Three-dimensional (3D) printing therapy was well-accepted by patients and offered a feasible, rapid, and automated approach to tailor made doses in hospital settings [32]. Further research and clinical trials exploring patient’s acceptance, response, and safety are necessary. 

The researchers estimated a gap of 17 years between a clinical innovation’s proven effectiveness and its routine adoption into the healthcare system [24]. There is a crucial need to collaborate between the 3D manufacturers, clinical practitioners, and regulatory bodies to develop strategies to accelerate the implementation of 3D printing in drug dispensing. This study is unique, as it for the first time assesses the knowledge and perceptions of the pharmacists along with elucidation of barriers faced by them in 3D printing implementation in their institutions.

## 5. Conclusions

Hospital pharmacists are critical for the future implementation of the 3D printing technique in pharmacy practice. Despite that, the hospital pharmacists who participated showed limited knowledge about the specific applications of 3D printing technique in drug dispensing. However, they showed a good perception about the concept of personalized medicine and how the 3D printing technique could provide a solution for the dispensing of tailored medicine based on patient needs. This knowledge gap and the good perception toward 3D printing technique among the practicing pharmacists’ call for continuous update of the learning resources to keep pace with the advances of pharmaceutical research. The cost of this technology, regulations, staff shortages, and the infrastructure were among the key barriers that might hinder the implementation of this technology. Therefore, to hasten the implementation of 3D printing technique in pharmacy practice, effective collaboration is needed to overcome these barriers. 

## Figures and Tables

**Table 1:** Socio-demographic characteristics of study participants.

Characteristic	Parameters	Number (*n* = 156)	Percent
Hospital type	Ministry of Health (MoH) Hospitals	80	51.2
	Other governmental Hospitals	61	39.1
	Private	15	9.6
Designation	Pharmacist	107	68.5
	Senior Pharmacist	38	24.3
	Consultant Pharmacist	11	7.0
Position	Non-administrative	100	64.1
	Administrative	56	35.8
Educational qualification	BSc pharmacy	64	41.6
	Pharm D	36	23.4
	MSc	39	25.3
	PhD	15	9.7
Number of years since completing the latest degree	<2	36	23.4
	2–4	23	14.9
	5–7	39	25.3
	8–10	31	20.1
	>10	25	16.2

## Data Availability

Data is contained within the article.
